# Clinical Conversations with His Private Classes at Demlit Dispensary, by L. Duncan Bulkley

**Published:** 1876-02

**Authors:** 


### Our Exchanges.

---

Clinical Conversations with his Private Classes at Demilt Dispensary, by L. Duncan Bulkley.—I have repeatedly called attention to the necessity of accuracy of diagnosis in diseases of the skin, as the first step toward correct treatment, and I have several times shown you how a slight misinterpretation of facts can construe a case into one of constitutional syphilis, whereas careful study and close observation have demonstrated how very grave would be the error and how inexcusable. The case before us affords a good illustration of this.

Case 1. *Papular Eczema of the Chest.*—James T., aged 37, a waiter, gives the following history: About two and a half years ago he had a chancre and buboes, which suppurated, since which time, he says, he has had falling of the hair, sore throat and rheumatism, and he comes, as so many patients will, impressed with the idea that the present eruption, which has existed nearly two years, is the result of venereal disease. Examining the chest, we find the centre of it, to the width of two hands-breadth, covered with a scaly, brownish-red papular eruption. Further examination shows that there are a few spots of the same on the head and one or two on the septum nasi.

The eruption here seen might be mistaken for several others, namely: tinea versicolor, psoriasis or papular syphilis. Tinea versicolor, sometimes wrongly called chloasma or liver spots, would not present so elevated an eruption, the color would be of a more tawny brown or yellowish, there would not be so many or so large scales, and it would not itch, as he tells us this does very considerably at times. Moreover, the eruption on the head and in the nose points to a constitutional disease, whereas you know that the tinea—or pityriasis—versicolor is a purely local one, due to the presence of a vegetable parasite.

Psoriasis would be probably found to exist elsewhere, as on the extremities, if this were that disease; it would not continue to itch, would have rather more scales, presenting the well-known micaceous appearance, and would not have had moist stages, as this has at times; nor would it affect the septum nasi.

Some would be led to conclude that the eruption was due to syphilis, considering that he gives a history of a genital sore followed by falling of the hair, sore throat and rheumatism. But it is just here that careful discrimination should be made, for we find that what he calls a chancre was followed by buboes which suppurated, a characteristic of chancroids and rare in the simple infecting sore of syphilis; we learn that the rheumatism was confined principally to the shoulders and to the soles of the feet, localities more commonly affected by ordinary or gouty rheumatism. The alopœcia seems to have been constant since then, whereas that caused by syphilis is generally transient; and finally, sore throat, when stated by the patient, is a very uncertain sign of syphilis. We discover, furthermore, that he has had no general eruption, nor indeed any other besides this one (which exists to a slight extent on the back).

Looking now at the eruption, it is seen to be made up of small papules, aggregated into patches of red, slightly inflamed skin, bearing evidence of itching in the numerous scratched papulæ and lines of excoriations. Now I know of no eruption caused by syphilis which resembles this, or which would be at all likely to attack the breast alone, in this form; nor would any syphilitic eruption maintain the same character for so long a time, or itch as this does.

The eruption has, however, all the appearances and history of papular eczema, and such in reality it is, and I think that we will find that it will disappear entirely under the following local treatment: Liquor picis Alkalinus, (R potass. causticæ ʒj picis liquidæ ʒij aq. destillatæ ʒv) full strength, to be rubbed on the parts, which are then to be covered and kept covered with this ointment; ℞. bismuth sub. nit. ʒj ung simplicis ℥j.

Case II. *Eczema of the Scrotum in a boy.*—James S., aged 8 years, was here for the first time three days ago, presenting the pitiable sight, not often seen in one so young, of eczema affecting the scrotum; the amount of suffering he has endured with it cannot be inferred from the present condition, and I will recall the state observed at his first visit. His history is as follows: When six months of age he had inflammation of the eye, and since he was two years of age has had an eruption on the skin, disappearing sometimes for awhile, but returning soon again. He has been free from cutaneous trouble from last spring until about six weeks ago, when an eruption made its appearance between the shoulders and on the chest, extending downwards since that time. About ten days ago it reached the scrotum and thighs, since which time the child's sufferings have been intense. There is no history of eruptions in the family, he being the eldest of three healthy children. There is no history of tubercular consumption or asthma in the family, but the father has rheumatism, and it prevails also in his family.

The boy is seen to be rather delicate, pale and thin. The skin of the back and abdomen is the seat of a papular eczema, bearing some marks of scratching, and on the inside of the thighs, extending two-thirds of the way down, an eruption is yet visible, papular, but with the elements much closer together than on the back and abdomen, and with some exudation from a few scratched spots. The present appearance of the scrotum is not striking, the skin being simply a little thickened, with a few remains of scratches on it; but when first seen, three days since, on Saturday, there was double the amount of the present thickening, the surface was cracked in many places, and evidences of exudation were present in scattered and light crusts. We can hardly appreciate the relief which he has obtained from the following treatment, namely: a soaking in a warm bath every night for fifteen or twenty minutes, made with potash, soda, and starch, thus: ℞. Potass. carb. ℥iv sodæ bi-carb. ℥ij div. in pulv. no iij. Use one for each bath, together with four tablespoonfuls of dry starch, boiled in about a quart of water. This, you will see, is a very small quantity of alkalies, but the patient having no long tub, must take a sort of sitz bath in an ordinary wooden wash tub). After carefully drying the parts without friction, this ointment was thoroughly applied: ℞. Ung. picis ℥ss Ung. simplicis ℥j. He was at the same time given internally, ℞. Liquoris arsenici chlor. ʒij pulv. rhei. sod. bi-carb. aa. ʒj aquæ menth. virid ℥iv. One teaspoonful three times daily. The alteration in the disease is so marked that I will make no change in treatment. The scrotum is now soft and almost natural looking, the treatment having enabled him to carry out my injunction not to scratch the parts.

Case III. *Herpes Zoster extending on to the fingers.*—The diagnosis of herpes zoster, zona, or shingles, should, as a rule, present but little of difficulty, but it is well to familiarize the eye with the appearance of the eruption. Here we have a very typical instance of the disease affecting the left upper thorax and arm. The girl, K. C., aged sixteen, of fair development and health, first noticed the eruption three days ago, having suffered, however, from some considerable neuralgic pain for a day or two previously, in the parts, which still remains, in a measure.

On the back of the left shoulder you see several patches of erythematous skin, with some papulæ and several vesicles just making their appearance. All these groups of efflorescences are embraced within a zone of about four inches width, upon the back, and about the same surface is occupied by it on the front of the chest. In this case, as the spinal nerves here implicated give off those innervating the arm, we find the disease leaving its true zonar location and following the nervous distribution upon this member. Here are some groups of vesicles on the back of the ulnar side of the upper arm, likewise on the flexor surface of the wrist, and in this instance there is even a bunch of vesicles on the ulnar aspect of the last phalanx of the middle finger, and here is a single vesicle on the ulnar side of the ring finger.

As I remarked before, well developed cases of shingles should offer little difficulty in diagnosis to one reasonably well informed in diseases affecting the skin, but errors not unfrequently happen either when the eruption is just appearing or when it presents an anomalous character. Very recently a patient of mine, when in a country city, was attacked by the severe unilateral neuralgia of the lumbar region, precursive to an attack of zona. The physician there gave him great alarm by affirming that he had kidney trouble. Internal remedies were given, external counter-irritants applied, and when the eruption began to make its appearance the doctor was non-plussed, and said that it was the "disease working to the surface;" the patient, however, thought the eruption was caused by the local applications. He came at once to the city, and was unspeakably relieved when the true nature of the skin trouble was explained, and the cause of the associated pain. In the present case, if the girl should, from delicate reasons, show a physician only the vesicles you see on the two fingers, and perhaps those on the wrist, the diagnosis would be very difficult if not impossible; hence the necessity of my oft-repeated instruction to ask first if the eruption shown is all that exists or has existed on the body; and if there is yet more, to insist, if at all practicable, in inspecting the whole. The distinctly unilateral character of the eruption of herpes zoster, the peculiar grouping of the vesicles (or perchance only yet papules) on erythematous bases, the distribution along nerve tracts, the burning and tingling with the eruption, and often the quite severe preceding or accompanying neuralgic pain are pathognomonic, and such a grouping of symptoms cannot be presented by any other disease of the skin. Let me throw out the hint, from the private case which I have mentioned, that whenever you are called to a patient with severe unilateral neuralgic pain, the suspicion that it may herald an eruption of zoster will not be far from right in many instances, and the prediction that an eruption may follow, might preclude much distress and anxiety when it did come.

The treatment of this case will be very simple; the neuralgia is on the wane, the patient strong and healthy, and I will simply direct that the whole affected surface be very perfectly dusted with powdered starch, and that a wide bandage of soft muslin, large enough to cover all the disease, be also thoroughly dusted with the starch, and then sewed around the patient, in such a way as to make an artificial covering, over which the clothing will slide. This bandage is not to be removed for a week or longer, till the vesicles are entirely dried, and perhaps the crust ready to fall off. This method of treatment is the simplest I know of, and efficacious; the relief it gives to the patient is immense, and I rarely order any other, even in private practice. There is no special call for medicine in this case, and she will only have a little rhubarb and soda mixture as a *placebo.*

A contrast to this case, in regard to the age of the patient and the form of development, is presented by this old man with the herpes zoster on the right arm only, now drying up, which I will briefly recall.

Case IV. *Herpes Roster affecting only the arm, sparing the body.* J. M., seventy-three years of age, a well-preserved and quite vigorous old man, complained greatly, when first seen, of the pain accompanying a zoster of the right arm, the pain being most marked in the thumb, and keeping him from sleep, as his wife testified. The remains of the eruption of vesicles and bullæ, perfectly characteristic of herpes, occupy most the internal or flexor surface of the arm, and extend very evidently on to the radial side of the thumb, to its tip. Near the shoulder the patches turn around the arm so as to occupy the dorsal aspect of the shoulder, but hardly extend above the end of the humerus, and the front and rear of the chest are spared.

What the cause of this rather anomalous arrangement in this case is I cannot say. The pathology of the disease is pretty firmly established, namely, nerve inflammation and infiltration, and several post mortem examinations have demonstrated that the ganglion on the posterior or sensory root of the spinal nerves, is the seat of well-marked inflammatory changes, and why the tract of skin between the spinal cord and the arm on the back, and the corresponding front part of the chest, are free from eruption in this case is impossible to conjecture, except on the theory that an idiopathic inflammation has arisen in some of the nerves themselves which compose the brachial plexus.

This patient complains loudly of the pain, and I will give him the phosphide of zinc combined with the extract of nux vomica, of each one-third of a grain in pill, every three hours, which I have found to control the pain wonderfully, as suggested by Dr. Ashburton Thompson. Locally the arm has been, and will continue to be, dressed with the powdered starch, as in the other case.

The neuralgia in this patient, you observe, is a prominent feature; this is generally the case in those past middle life, and you must be careful how you predict its rapid disappearance, for sometimes it is a most distressing symptom, and gives physician and patient much trouble. See how some of these vesicles are drying into hard crusts; if these are pulled off troublesome ulceration may occur in the aged.

Case V. *Erythema Simplex, of the variety circinatum.*—This woman, aged about fifty, presents herself with an eruption which it is impartant for us to study from a negative point of view. She says that a few days ago she noticed the development of red spots upon the forehead, accompanied with burning, tingling, and slight itching. When she first entered the room you noticed that I examined the parts very closely, and doubted somewhat the existence of any skin lesion. She had been sitting by the stove in the ante-room, after coming in from the very cold outer air, and the whole forehead appeared uniformly reddened, and exhibited nothing different from what may be observed on many patients with other troubles who have been similarly exposed to heat and cold; the redness disappeared momentarily on pressure. But now, after sitting here in a cooler room for five minutes, the eruption for which she comes is distinctly visible.

We now perceive a collection of erythematous patches, varying in size and shape; mostly circular, from one-quarter to one inch in diameter, extending across the forehead from one zygomatic arch to the other, reaching, some of them, to the hairy scalp above; others to the eyebrows, and encroaching, to a slight extent, on the upper part of the right cheek. The centre of these larger patches, you see, has already paled, so that they appear as rings, while the smaller ones present an uniform redness. The eruption is found to be entirely erythematous, disappearing under pressure, the redness returning almost instantly after removing the finger; there is no thickening of the skin, although, as the finger is passed lightly over the surface, a very slight elevation is barely perceived, congestive in character; there is no exudation from the surface, no scales or crusts, and there is no appreciable itching.

I have called this eruption erythema simplex, for it is the simplest form of congestive disturbance we have; it is here, however, not due to mechanical cause, as when from heat, cold, friction, irritants, and the like, but is undoubtedly a reflex affair, much like urticaria, and dependent upon digestive, or uterine, or ovarian derangement. But in this case the woman is about fifty years of age, and has passed the menopause, and sexual influences are less probable; whereas, her tongue is pale and is slightly coated, the bowels sluggish; she gives a rheumatic history, and we may therefore readily attribute the eruption to such diathetic causes as operate in giving rise to many of the congestive lesions of the skin, than which I doubt if we can approach nearer to a pathological etiology in the present state of medical science, the exciting cause being probably some faulty digestion. At any rate, I feel pretty confident that she will be freed from the trouble in a very few days, by the use of what you hear me prescribe often as Sartin's mixture (although I cannot vouch that the formula is exactly his), which is slightly laxative, contains magnesia and iron, as follows: ℞. Magnesiæ sulphat. ℥j, ferri sulph. ʒj, acid. sulph. aromat. ʒiv, tinct. gent. ℥j, aquæ ad. ℥ij. Sig. One teaspoonful after meals.

A moment more may be profitably spent in considering the elements in the diagnosis in this case. The only other affections for which this could be mistaken are, erysipelas, eczema, and urticaria. In erysipelas the eruption would be more diffused and confluent, and not in the form of circles and separate patches; the surface would be more red and shiny, here it is pinkish; it would have made greater progress in three days, and there would be more heat in the part, with, probably, constitutional disturbance; she appears to have none. Were it eczema, itching would be present to a greater degree, there would be exudation, either in or on the skin, some unevenness of surface, and the eruption would not thus entirely disappear on pressure. Urticaria would likewise give more itching, would certainly have affected other parts of the body as well, and its elements would have been more transitory, the individual efflorescences of urticaria are very short lived, these have been constant; urticaria is more elevated and its wheals more irregular in shape. I know of nothing in syphilis with which this could be confounded, and acne rosacea, which exists in the minds of some as simply an erythematous disease, not present this history; it seldom attacks the forehead alone, is of a deeper hue, and presents either true acne elements or enlarged blood vessels.

(Note.—The patient returned after four days, with all traces of the eruption vanished; no other treatment was employed, internal or external.)

Case VI.—*Artificial Eczema of forehead, caused by an irritating hat lining.*—The case of this patient, J. S., aged twenty-seven, is worth noticing as an instance of the local irritation going on, even to in inflammation of the skin (dermatitis), and ending in eczema, from agencies which are seldom looked for. This man for ten months has had an eruption across the forehead, just where the band of the hat presses; it began with the wearing of a certain new hat, and has remained pretty much the same ever since. Here is a band of red, itchy skin, about an inch in width, which you can see is infiltrated, because it presents some elevation, but which is more evident on pinching a fold of it between the thumb and finger, and comparing it with a similar portion of healthy skin from about the same region, the difference being very striking. We have here now all the elements of chronic eczema; he further tells us that it "waters" at times, meaning, of course, that there is exudation when it is irritated; and this patch had its origin in the local irritation of the hat-band, possibly from the dye, possibly from some fault in the tanning process, but, from the fact of the rarity of such cases, and from the persistency of the disease, I am justified, I think, in calling it no longer an artificial dermatitis, but an eczema.

He will receive the following ointment, with directions to rub it firmly into the part, night and morning; first with the fingers, and later on with a bit of flannel, as the skin gets accustomed to it, and to keep the surface coated with the ointment as much of the time as possible: ℞. Liquoris picis alkalin. ʒj, zinci. oxidi. ʒj, Ung. aquæ. Rosæ. ℥j. M. We will direct him also to line his hat with old linen.—*Archives of Dermatology.*

